# The development of a Simplified, Effective, Labour Monitoring-to-Action (SELMA) tool for Better Outcomes in Labour Difficulty (BOLD): study protocol

**DOI:** 10.1186/s12978-015-0029-4

**Published:** 2015-05-26

**Authors:** João Paulo Souza, Olufemi T Oladapo, Meghan A Bohren, Kidza Mugerwa, Bukola Fawole, Leonardo Moscovici, Domingos Alves, Gleici Perdona, Livia Oliveira-Ciabati, Joshua P Vogel, Özge Tunçalp, Jim Zhang, Justus Hofmeyr, Rajiv Bahl, A Metin Gülmezoglu, On behalf of the WHO BOLD Research Group

**Affiliations:** Department of Social Medicine, Ribeirão Preto Medical School, University of São Paulo, Av. Bandeirantes, 3900 Ribeirão Preto, SP Brazil; GLIDE Technical Cooperation and Research, Ribeirão Preto, SP Brazil; UNDP-UNFPA-UNICEF-WHO World Bank Special Programme of Research, Development and Research Training in Human Reproduction, Department of Reproductive Health and Research, World Health Organization, Avenue Appia 20, 1201 Geneva, Switzerland; Department of Population, Family and Reproductive Health, Johns Hopkins Bloomberg School of Public Health, 615 N Wolfe St., Baltimore, MD 21205 USA; Department of Obstetrics and Gynaecology, Makere University, Kampala, Uganda; Department of Obstetrics and Gynaecology, College of Medicine, Ibadan, Nigeria; Shanghai Jiaotong University, School of Medicine, Shanghai, 200030 People’s Republic of China; Department of Obstetrics and Gynaecology, Frere Maternity Hospital, P Bag X9047, East London, South Africa; Department of Maternal, Newborn, Child and Adolescent Health, World Health Organization, Avenue Appia 20, 1201 Geneva, Switzerland

**Keywords:** Partograph, Labour monitoring, Decision-support tool

## Abstract

**Background:**

The partograph is currently the main tool available to support decision-making of health professionals during labour. However, the rate of appropriate use of the partograph is disappointingly low. Apart from limitations that are associated with partograph use, evidence of positive impact on labour-related health outcomes is lacking. The main goal of this study is to develop a Simplified, Effective, Labour Monitoring-to-Action (SELMA) tool. The primary objectives are: to identify the essential elements of intrapartum monitoring that trigger the decision to use interventions aimed at preventing poor labour outcomes; to develop a simplified, monitoring-to-action algorithm for labour management; and to compare the diagnostic performance of SELMA and partograph algorithms as tools to identify women who are likely to develop poor labour-related outcomes.

**Methods/Design:**

A prospective cohort study will be conducted in eight health facilities in Nigeria and Uganda (four facilities from each country). All women admitted for vaginal birth will comprise the study population (estimated sample size: 7,812 women). Data will be collected on maternal characteristics on admission, labour events and pregnancy outcomes by trained research assistants at the participating health facilities. Prediction models will be developed to identify women at risk of intrapartum-related perinatal death or morbidity (primary outcomes) throughout the course of labour. These predictions models will be used to assemble a decision-support tool that will be able to suggest the best course of action to avert adverse outcomes during the course of labour. To develop this set of prediction models, we will use up-to-date techniques of prognostic research, including identification of important predictors, assigning of relative weights to each predictor, estimation of the predictive performance of the model through calibration and discrimination, and determination of its potential for application using internal validation techniques.

**Discussion:**

This research offers an opportunity to revisit the theoretical basis of the partograph. It is envisioned that the final product would help providers overcome the challenging tasks of promptly interpreting complex labour information and deriving appropriate clinical actions, and thus increase efficiency of the care process, enhance providers’ competence and ultimately improve labour outcomes.

Please see related articles ‘http://dx.doi.org/10.1186/s12978-015-0027-6’ and ‘http://dx.doi.org/10.1186/s12978-015-0028-5’.

**Electronic supplementary material:**

The online version of this article (doi:10.1186/s12978-015-0029-4) contains supplementary material, which is available to authorized users.

## Background

Labour complications are an important cause of mortality, morbidity and long-term disabilities for both mothers and babies, particularly in under-resourced settings [1]. A substantial proportion of these severe outcomes occur in the community or at primary level health facilities, where women often deliver alone or assisted by semi-skilled birth attendants. In many health facilities, delays in implementing appropriate interventions often result in severe adverse outcomes for mothers and babies. Applying interventions when they are not medically indicated (e.g. caesarean sections, labour induction/augmentation) can also lead to iatrogenic complications, avoidable suffering and inequitable distribution of limited resources [2,3].

The identification and appropriate management of women at high risk of labour complications (including at the first contact with the health system), careful monitoring throughout labour and childbirth, timely use of effective interventions (e.g. labour augmentation, assisted vaginal delivery and caesarean section) together with appropriate neonatal resuscitation would avert most of the avoidable intrapartum related maternal and perinatal deaths. The partograph is the main tool to track the progress of labour and its use is generally regarded as essential for appropriate labour monitoring. However, a major obstacle for improving birth outcomes is that intrapartum monitoring with the partograph is a time consuming task and it is known that partograph is either not used or incorrectly used in most low and middle-income countries [4-7]. Time constraints, staff shortage, lack of knowledge and negative attitude among healthcare providers were some of the obstacles noted to hinder appropriate use of the partograph [8,9]. In busy labour wards in under-resourced settings, overloaded health professionals often struggle to provide appropriate monitoring for all women. Another major limitation is that making sense of all relevant information to derive appropriate actions is not always straightforward. Therefore, a novel approach to improving labour monitoring and management is urgently needed.

### Rationale for developing a new tool for labour monitoring

General curves of normal labour progress were developed by Friedman using graphico-statistical analyses in the 1950’s. Friedman reported that cervical dilatation follows a sigmoid curve for most women and his work is considered the basis of the “1 cm/hr rule”. This rule is commonly used in clinical settings as the reference slowest-yet-normal cervical dilatation rate during active first phase of labour (from the onset of maximum slope to complete dilatation) [10]. Building on Friedman’s original work, Philpott and Castle developed the partograph in the early 1970’s [11-13]. The partograph is a graphic tool displaying length of labour in hours (x -axis) and cervical dilatation in centimetres (y-axis). Key features of this tool are the alert and action lines, which are meant to function as triggers of interventions during labour. The alert line is straight/linear and represents a cervical dilatation rate of 1 cm/hr. The action line is parallel to the alert line and it is displayed four hours to the right of the alert line. Thus, the partograph has an underlying algorithm aimed at identifying women who are likely to present labour-related poor outcomes. Also anchored in the “1 cm/hr rule”, O’Driscoll and colleagues proposed during the 1970’s the Active Management of Labour (AML) as a package of interventions aiming at reducing the proportion of women with labour progressing at cervical dilation rates lower than 1 cm /hr [14,15]. A product of that era, the partograph, has since been promoted as an essential tool for assessing labour progress.

However, despite the efforts of major international organizations to increase the use of the partograph in labour monitoring, the rate of appropriate use of the partograph for labour management is disappointingly low [4-7]. This could partly be because obtaining the information needed in a timely manner and plotting it into the partograph can be a complex task, particularly in busy, understaffed units. Another limitation is that interpreting the information available and deriving appropriate clinical actions can be a challenging task for many health providers. Additionally, evidence of positive impact of the partograph in labour-related health outcomes is lacking [16] and there is mounting evidence that the pattern of labour progression among low-risk women with spontaneous onset of labour differs substantially from Friedman 1950’s reports [10]. According to Friedman studies, only 10% of women in labour would cross the “alert line”, but Philpott and Castle had observed 21.8% of African women crossing the “alert line” [10-13]. Similarly, the 1994 WHO partograph study showed that 30.9% of women in labour crossed the “alert line” [17]. More recently, Orji and colleagues, studying Nigerian women in labour, found that 34.8% of nulliparas crossed the “alert line” and 18.5% crossed the action line [18]. Many factors could explain the existence of different patterns of labour and delivery progress (e.g. anatomical differences in the pelvic configuration). Thus, it seems that the straight, linear, 1 cm/hr cervical dilatation rate may be inappropriate as a universal rule and unrealistically fast for many women; and its general application may lead to unnecessary interventions during labour [19-21]. Contemporary obstetric attitudes and practices have changed since Friedman’s work and currently, there are new statistical and computational techniques that could allow the development of a tool that is able to provide customized guidance to health providers tracking labour progress. Technically, the partograph is a static, linear and bi-dimensional classifier that has the challenging task of discriminating women that are likely to experience labour complications from those that are not. Artificial intelligence (AI) techniques could enable the development of more sophisticated and potentially more effective classifiers, using dynamic, non-linear, multidimensional mathematical models. AI applications currently perform complex tasks and are now commonplace consumer items, including applications in medicine, such as automated external defibrillators and specialized clinical decision support systems (mostly in oncology, abdominal pain diagnostics, and internal medicine). We have carried out a review of the literature and did not identify an AI tool for labour management that could be used in under-resourced settings.

### WHO Better Outcomes in Labour Difficulty (BOLD) project

The World Health Organization has initiated a project to address the quality of facility-based intrapartum care in under-resourced settings [22]. The goal of this project is to accelerate the reduction of intrapartum-related maternal, fetal and newborn mortality and morbidity by addressing the critical impediments in the process of labour care and establishing the desired connection between the health system and the community. This project seeks to achieve this goal through the development of an evidence-based and easy-to-use labour algorithm and innovative tools that create community demand for quality intrapartum care.

### Simplified, Effective Labour Monitoring-to-Action tool (SELMA)

In order to address labour monitoring constraints (i.e. time-consuming and complex monitoring, unclear link between monitoring and action, making sense of complex information), the concept of a Simplified, Effective, Labour Monitoring-to-Action (SELMA) tool has been developed. SELMA will form the basis for the development of an optimal labour care algorithm as informed by a cohort study of women delivering in facilities and formative research around provider and health system issues relating to labour management. It is envisioned that the tool will alleviate the burden of health professionals during labour, foster optimal labour management and optimize task shifting by supporting decision-making of less specialized health professionals. SELMA would help providers overcome the challenging tasks of promptly interpreting complex labour information and deriving appropriate clinical actions, and thus increase efficiency of the care process, enhance providers’ competence and ultimately improve labour outcomes. This protocol describes the quantitative research required for the development of a simplified, effective monitoring-to-action algorithm and tool for sub-Saharan African women in labour. The formative research protocol has been published separately [23].

### Study objectives

Considering that the partograph has underlying algorithms and SELMA will embed algorithms designed to identify women who are likely to develop labour-related poor outcomes (and point to actions to prevent them), the primary objective is to identify the essential elements (including thresholds and interactions) of intrapartum monitoring that trigger the decision to use interventions aimed at preventing poor labour outcomes in order to develop a simplified, monitoring-to-action algorithm for labour management. Secondary objectives are to (i) compare diagnostic performance of SELMA and partograph algorithms as tools to identify women likely to develop poor labour-related outcomes and; (ii) To explore the development of modern curves of normal labour progress for sub-Saharan African women.

### SELMA conceptual framework

The main goal of this project is to develop a viable alternative to the partograph. SELMA will be a tool designed to:

1. Acquire data (through health providers) about the characteristics of women in labour, their labour progression and the setting where labour is taking place;
2. Keep records and conveniently display intrapartum monitoring-and-care information;
3. Determine the probability of adverse or favourable birth outcomes if an intrapartum related intervention is performed or not performed, considering the individual characteristics of women in labour, their labour progression and setting where labour is taking place;
4. At each point in time, identify the course of action that prevents the main adverse outcome of interest and maximizes the likelihood of good outcomes, based on the individual characteristics of women in labour, their labour progression and setting where labour is taking place;
5. Report this information to the health providers.

**Figure 1:**
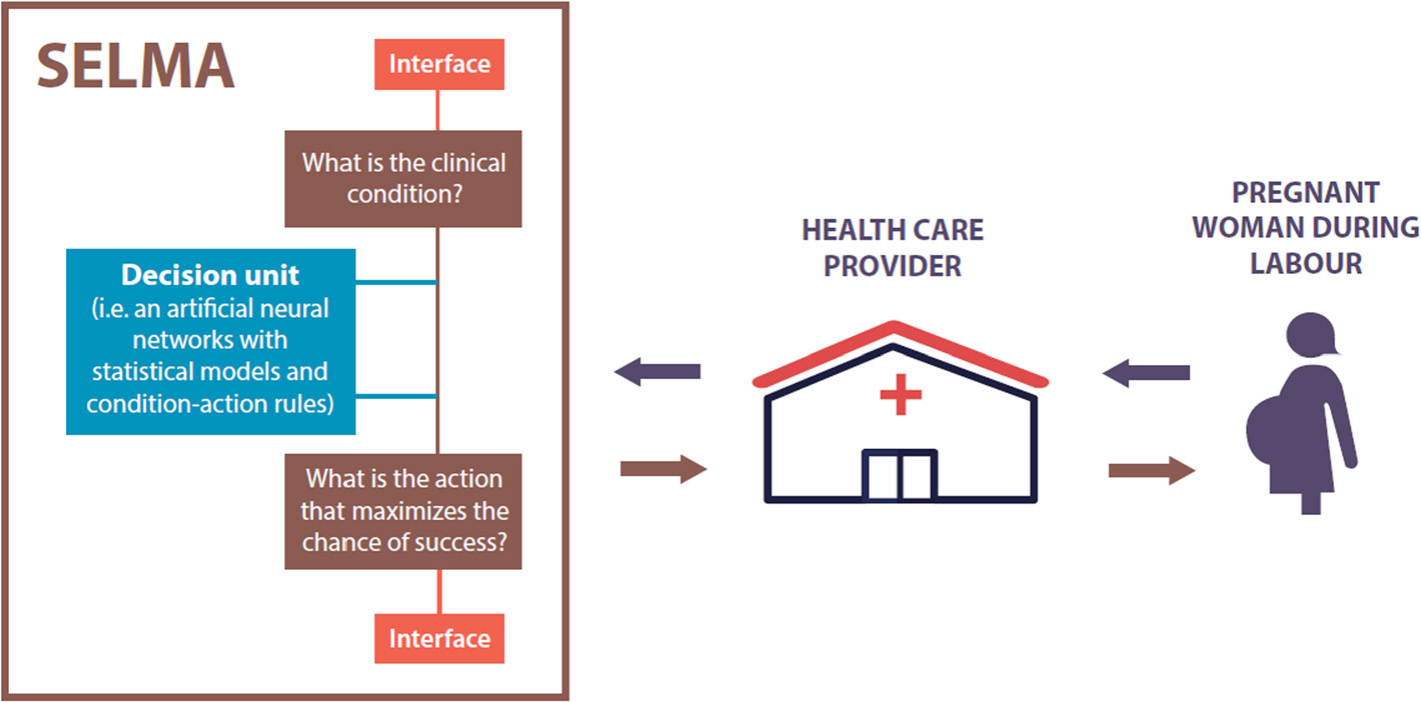
Diagram of how SELMA will work.

**Figure 2:**
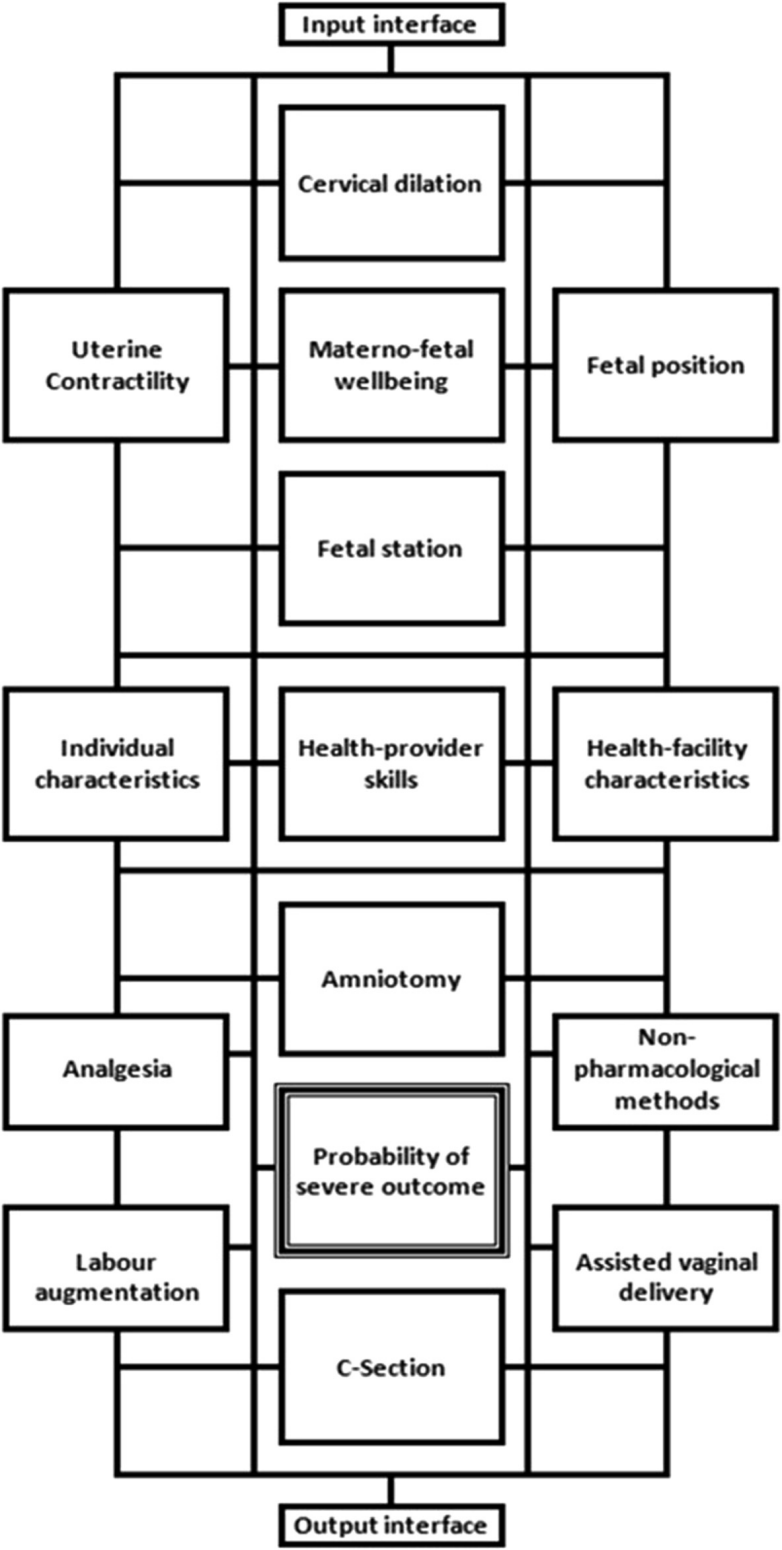
A preliminary diagram of SELMA decision unit.

A diagram representing how SELMA will work is presented in the Figure 1. In the core of SELMA there will be a set of interconnected mathematical models (network) that will be integrated into an evidence-based clinical algorithm. A key function of this decision unit will be to assess the clinical situation and prompt health care providers to either allow labour to progress with routine monitoring or perform non-medical or medical interventions, such as intensified clinical assessments, changes in maternal position and mobilization, amniotomy, pharmacologic augmentation, caesarean section/instrumental vaginal delivery. Figure 2 presents a more detailed diagram of SELMA decision unit.

In order to build the SELMA decision unit, this project aims to answer the following research question: in women in labour in African health facilities, what are the elements of intrapartum monitoring (including thresholds and interactions) that trigger the decision to use interventions aimed at preventing poor labour outcomes? In other words, this project aims at determining the relationship between candidate predictors, intrapartum care interventions and labour outcomes. Candidate predictors will be obtained from patient demographics, clinical history, physical examination, characteristics of labour progress, and previous interventions (e.g. labour augmentation or amniotomy). The candidate predictors will be clearly defined and reproducible to maximize generalizability and applicability of study results to practice. In addition, the candidate predictors will be measured using methods applicable to daily practice. We will avoid overly specialized or high-tech measurement techniques as this may limit the applicability of the models. Each of the candidate predictors selected will be available or could be easily made available in an average African district health facility.

## Methods

### Study design

A prospective cohort study is proposed. This study design was selected because SELMA development will require the development of a set of integrated prognostic models related to labour and childbirth. A prospective cohort study minimizes selection and reporting bias to the greatest extent possible thereby representing the strongest design with the greatest likelihood of providing a clear and accurate assessment of the relationship between candidate predictors (described below) and composite outcome of interest (i.e. intrapartum related death and morbidity).

### Study sites

This study will be conducted in eight health facilities in Nigeria and Uganda (four facilities from each country). Inclusion criteria for health facilities are: a minimum of 1,000 deliveries per year, the major health care facility in its region, and not a primary health care unit), Intrapartum care provision by skilled birth attendants and stable access to caesarean section, augmentation of labour, assisted vaginal delivery and good intrapartum care practices (e.g. intermittent fetal monitoring, respectful maternity care, good midwifery care).

### Study participants

#### Participants inclusion criteria

All women admitted for vaginal birth with single live fetuses during the first stage of labour (both in latent phase or early active phase) will comprise the study population. This includes women undergoing induction of labour and those with spontaneous labour onset presenting at cervical dilatation of ≤6 cm. Women will be considered for inclusion whether or not they primarily receive antenatal care and plan to deliver at the participating hospital.

#### Participants exclusion criteria

Women with any of the following conditions will be excluded from the study. Absence of an identifiable fetal heart sound at hospital admission (presumed intra-uterine fetal death); advanced first stage of labour (≥7 cm cervical dilatation); multiple pregnancy; gestational age less than 34 weeks (i.e. 33 weeks and 7 days); elective C-section; pre-labour C-section; indication for emergency C-Section or laparotomy on admission; attempted induction of labour, but no labour achieved, false labour, non-emancipated minors without a guardian; women who are not capable of giving consent due to labour distress or any health problem(s), such as obstetric emergencies (e.g. eclampsia) or mental disorder.

#### Participant recruitment

Assessment of study eligibility and recruitment of participants will be carried out by trained research nurses, who will approach women for consent for participation in the study at hospital admission except when they meet any of the exclusion criteria listed above.

### Outcomes of interest

The main outcome of interest in this study is intrapartum-related perinatal death and morbidity. This is a composite outcome comprising intrapartum-related stillbirths (i.e. “fresh stillbirths”), very early neonatal deaths (i.e. neonatal death taking place in the first 24 hours of birth) and neonates with Apgar score <6 at 5 minutes of birth (i.e. Apgar score that best indicates neonatal asphyxia with possible serious adverse consequences). Table 1 presents prevalence data concerning main outcomes of interest observed in Nigerian and Ugandan Hospitals during the three months of data collection of the WHO Multicountry Survey of Maternal and Newborn Health [24]. The composite outcome is limited to these three conditions as they represent critical adverse outcomes with huge global burden where improvement in the process of intrapartum care could make a difference. In addition, they can be easily and objectively measured and thus reduce the potential for detection bias in the context of a multicenter study setting. More importantly, the three adverse newborn outcomes are likely to share the same set of predictors as they are logically related in the pathway between pathological insults and death.

**Table 1:** **Intrapartum-related perinatal death and morbidity in Nigeria and Uganda**

	**Nigeria**	**Uganda**	**Total**
	**n**	**n**	**N (%)**
Hospitals	21	21	42
All Births	12,352	10,923	23,764 (100%)
Intrapartum Fetal Deaths	489	196	685 (2.9%)
Very Early Neonatal Death	70	62	132 (0.6%)
Apgar Score <6 at 5’	240	147	387 (1.6%)
Intrapartum Related Perinatal Death & Morbidity*	762	376	1138 (4.8%)

*A total of 66 neonates with Apgar Score < 6 at 5’ died in the first 24 hours postpartum and are also counted as “Very Early Neonatal Deaths”.

A secondary outcome of interest is a “good perinatal outcome”. This is a composite outcome that describes healthy neonates and defined as (i) neonates born alive, (ii) with Apgar score >7 at 5 minutes, (iii) without death or severe morbidity at 24 hours of birth or discharge (whichever comes first).

### Sampling and allocation

Considering that countries had been previously selected based on the good performance of their local research teams in the WHO Multicountry Study on Maternal and Newborn Health and the availability of funds to conduct research in those countries, a two-stage sampling strategy will be used to sample health facilities and individuals to participate in this study. In the first stage, convenience sampling will be used to identify health facilities fulfilling the inclusion criteria for health facilities. The candidate health facilities will be identified by the local principal investigators and confirmed by the WHO coordinating unit after site visits. In the second stage, trained research staff operating at the participating health facilities will invite all women admitted for vaginal birth and not presenting with any of the exclusion criteria above to participate in the study. This cohort study has only one study group (group allocation strategies are not applicable).

### Sample size calculation

In order to achieve the main objective of this project, a total of 7,812 women in early labour are needed. The sample size calculation was based on the number of candidate predictors (N = 20 (maximum number)), the minimum number of outcomes per predictor considered for model development and validation (M = 15; 10 in the training set and 5 in the validation set); I = incidence of the main outcome of interest (I = 4.8%) and a margin of error (ME = 25%, also accounting for the clustering effect).

$$ \mathbf{Sample}\ \mathbf{size} = \left(\left(N \times M\right)/I\right) \times \left(1+ ME\right) = \left(\left(20 \times 15\right)/0.048\right) \times \left(1 + 0.25\right)=\mathbf{7},\mathbf{812} $$

The incidence of the main outcome of interest was based on data derived from the WHO Multicountry Survey on Maternal and Newborn Health (WHO MCS) in Nigeria and Uganda (Table 1).

The number of health facilities was determined based on the average annual number of births of district/secondary level hospitals that participated in the WHO Multicountry Survey on Maternal and Newborn Health for Nigeria and Uganda and a recent census carried out among candidate health facilities. Considering a 6-month data collection period and that only 50% of the women are in early labour, eligible and willing to participate a total of eight health facilities (4 per country) will take part of this study (participating hospitals are expected to recruit 1,000 women on average).

### Drugs and devices

Doptones will be used to assess fetal vital status at arrival and perform intermittent fetal monitoring during labour and delivery. All participating health facilities will receive Doptones and training to use them in order to standardize fetal heart rate assessment across participating hospitals.

### Innovation in service delivery

All women participating in this study will need to have the fetal vital status determined at hospital admission using a Doptone device. Doptones will be used to perform intermittent fetal monitoring during labour and delivery. The use of this device may represent an innovation in service delivery for some hospitals. The coverage of fetal vital status monitoring at admission will be one of the study protocol compliance indicators.

### Admission procedure

In each participating health facility, trained research nurses will screen all women admitted for vaginal birth using the screening form (Section A of the data collection form; See Supplementary Additional file 1). Once the eligibility of the women to participate in this study is determined, the research nurse will invite the potential participant to join the study and seek her individual consent using the individual consent form.

### Data collection procedures

Data will be gathered continuously for a period of 6 months at each facility. At each facility, research assistants will be trained to perform data collection and distributed to ensure coverage of typical hospital shifts. Through daily visits to the labour ward, delivery room, postnatal ward, and neonatal intensive care unit, the research assistants will continuously review the medical records of all recruited women and obtain information (if needed) from the attending staff in order to extract information required to complete the study forms. Research nurses will ensure that data extraction covers the three process levels of intrapartum care that are relevant to the study objectives i.e. hospital admission, labour and childbirth process, and postnatal period/hospital discharge). Data collection will start at hospital admission and end in the event of maternal death, transfer or hospital discharge. If the woman dies after a live baby has been born, data collection of infant data will be carried out until intra-hospital infant’s death, transfer or hospital discharge. Where a research assistant is a staff of the participating institution (e.g. a nurse), he/she will only collect data outside his/her routine working hours (i.e. data will not be collected by any staff at a time when such staff is also providing hospital care).

A hospital coordinator will facilitate and oversee the data collection process and training of local research staff, conduct training of existing hospital staff on adequate documentation of labour events, and transfer completed data collection forms to the country coordinator.

### Follow-up procedures

Data will be collected during hospital stay only. Data collection will start at hospital admission and will end in the event of maternal death, transfer or hospital discharge (see further details above). No post-discharge follow-up will take place.

### Criteria for discontinuation of a participant

Women who had initially given consent and later decline to continue participation will be discontinued from the study.

### Study instruments

This study will use a set of forms that will enable data collection at individual and facility level. The data management and analysis team developed draft forms in collaboration with the WHO study coordination unit and the country principal investigators. These draft forms were reviewed by the study coordinators and study steering committee. Based on these reviews, relevant changes and amendments were made to the forms, which were converted to advanced drafts. The advanced drafts were pilot-tested in a convenient sample of women in labour in one hospital of each country. The pilot-test generated additional changes to the forms. A second round of revisions by the local teams in each country was carried out during a training workshop. Once the forms were finalized, they were produced and dispatched to the participating hospitals.

For the purpose of this study, four sets of information will be collected.

1. The static/fixed information that will be collected at hospital admission, just after recruitment. This is essentially the information that will not change during labour (e.g. maternal demographics and past obstetric characteristics)
2. The dynamic information that is normally included as part of the partograph (fetal heart rate, status of the membranes, characteristics of liquor, cervical dilatation, station of the presenting part, uterine contractions, oxytocin augmentation and rate of oxytocin administration, use of analgesia, IV fluids, temperature, pulse and blood pressure, and urine assessment). Most of the information related to labour progress is part of the partograph and will be collected as recorded by the labour attendants in the traditional way that the partograph is completed.
3. The dynamic information that is not part of the partograph. This information will provide information on the implementation of specific effective practices and clinical observations that are not traditionally captured by the partograph. This information will be collected as the partograph information is collected and will be limited to small amount of data, essentially related to a) provision of social support, b) adopted maternal position and mobilization during labour and childbirth, c) oral food or fluid intake, and d) maternal wellbeing (e.g. hydration status).
4. Maternal and infant outcome data.

This information will be recorded on paper forms for individual study participants, and will include the following sections:

- **Participant Eligibility:** this form will confirm the eligibility of individual woman to participate in this study
- **Maternal Admission Characteristics:** this form will gather data on the social, demographic, anthropometric, obstetric and medical characteristics of study participants at hospital admission. These data will include maternal age, number of pregnancies including the index pregnancy (gravidity), previous births (parity), marital status, educational level completed, ethnicity, weight, height, use of prenatal care in index pregnancy, previous caesarean sections, number of previous abortions (either induced or spontaneous), presence of any previous uterine surgery, pre-existing medical problem(s) such as hypertension and diabetes, onset of labour (induced or spontaneous), physical findings on general examination (anaemia, fever, vital signs) and abdominal examination (frequency and intensity of uterine contractions, symphysis-fundal height, fetal movements, lie, presentation and position, fetal heart rate, and vaginal examination (cervical effacement [degree of shortening], cervical consistency, cervical dilatation, status of amniotic membranes, and characteristics of liquor if membranes are ruptured, descent of the presenting part relative to the level of ischial spines.
- **First Stage of Labour Events:** This form will collect data on the multiple assessments and interventions performed during the first stage of labour. These will include standard labour progress assessments (uterine contractions, fetal heart rate, characteristics of liquor, cervical dilatation, descent of the presenting part, amniorrhexis, presence or absence of caput succedaneum or molding), maternal clinical and laboratory observations (pulse, temperature, blood pressure, urine assessment), and specific interventions performed during the course of labour with aim of improving labour experience and outcomes (use and timing of analgesia, intravenous fluid administration, oral fluid intake, ambulation, companionship, oxytocin augmentation and rate of infusion, assisted vaginal delivery, and caesarean section).
- **Second Stage of Labour Events:** This form will collect data on the multiple assessments and interventions performed during second stage of labour.
- **Indication and Timing of Selected Interventions:** This form will collect additional data concerning the use of selected intrapartum care interventions, including indications, reasons for delays, decision-intervention time (e.g. decision-incision time for caesarean section) and adverse effects/outcomes related to these interventions.
- **Labour Outcome Data:** This form will collect maternal and neonatal outcomes and data related to the immediate postpartum period until hospital discharge. Specifically, data will be collected on maternal and perinatal intrapartum related death and morbidity. This will include labour obstruction, uterine rupture, dystocia-related severe maternal morbidity or death, intrapartum-related fetal death, intrapartum-related very early neonatal death (<24 hours of birth), Apgar score < 6 at 5’ min, neonatal encephalopathy, and neonatal admission to intensive care unit.

### Project management

The project management will include coordination and execution of the following activities which require administrative and clinical research input. The BOLD Project Steering Group will oversee the progress of the study, provide technical guidance and make policy decisions related to the conduct and implementation of the study. The Project Steering Group is made up of project staff at WHO, and lead investigators from University of São Paulo, Brazil; Makarere University, Uganda; University of Ibadan, Nigeria; and M4ID, Finland. The project will also receive technical advice regarding its implementation from a Technical Advisory Group (TAG) - a multistakeholder group comprising of experts in epidemiology, clinical obstetrics, midwifery, health system, service design, information technology, and programme implementation from both high and low-income countries.

### Preparation for the study

- Site visits before study starts: standardization of study procedures; procurement of study materials; verification of recruitment rate.
- Discussion of data collection logistics with collaborators.
- Establishment of communication procedures between the centres.
- Designation of Data Management and Analysis Committee
- Finalization of the protocol together with the local research teams
- Submission of proposal to relevant ethics committees.
- Organization of interim and final collaborators meetings.
- Preparation of data collection forms and consent forms.
- Preparation of Manual of Operations.
- Purchase and delivery of Doptones and other supplies to participating hospitals
- Conducting training workshops and eligibility drills with simulated and real cases

### Data processing and system preparation

On-line data entry and management will be coordinated by the Data Management and Analysis unit at the University of São Paulo, Brazil. Data analysis and interpretation will be done jointly between the Data Management and Analysis team at the University of São Paulo, and the Project Steering Group.

### Conduct of the study

#### Coordination activities

- Site visits to monitor study progress.
- Communication with local investigators to monitor trial progress.
- Communication with the data monitoring committee of the trial.

#### Data management and statistical analysis

- Data entry
- Data validation and production of queries
- Correspondence between coordinating centres and the centres related to queries.
- Monitoring reports: recruitment, loss to follow-up, completeness of data for main outcomes.
- Statistical and computational analysis: interim analyses, final analysis.

### Data quality assurance

Data collection forms, a manual of operations and a study database will be developed. Study data will be collected and managed using REDCap electronic data capture tools hosted at the Ribeirão Preto Medical School, University of São Paulo, Brazil. REDCap (Research Electronic Data Capture) is a secure, web-based application designed to support data capture for research studies, providing: 1) an intuitive interface for validated data entry; 2) audit trails for tracking data manipulation and export procedures; 3) automated export procedures for seamless data downloads to common statistical packages; and 4) procedures for importing data from external sources (http://project-redcap.org/). Data will be collected using paper data collection forms and then entered into REDCap. Aiming at complete and accurate data, a visual inspection of the form will be carried out before data entry; automated rules for detecting data inconsistencies or discrepancies will also be integrated. Data collection will be carried out by trained data collectors and will start at recruitment, e.g. time of admission and finish at the hospital discharge. Data on candidate predictors collected prior to the outcomes of interest will be used for analyses. The majority of the facilities has participated in the Global Survey/Multicountry Survey Project and has experience in facility-based studies. Online data entry system will minimize the data entry errors and facilitate monitoring and quick resolution of queries and missing data. A manual of operations will be developed to minimize the need for judgement and interpretation by the data collectors. The manual of operations will include a description of the study in general terms, emphasize the importance of complete and accurate data, and foster the standardization of data collection. The data collection tools will be reviewed by other researchers and pre-tested on a convenient sample of records and clinical settings. Reviewers will note their individual experience with both the definitional criteria and the time taken to collect and record data. Based on the final pre-test, revisions will be made to both data collection instruments. There will be training workshops at country-level. Routine hospital data about the total number of women admitted to the facility and delivering at the facility will be monitored and compared to the study data. Validity cross-checks will be performed. In addition, random cross-checks of 1% of the forms will be made to ensure that entered data correspond to the woman in question. The responsible hospital staff member will maintain a problem log book to document unanticipated problems. Technical questions encountered in the field will be resolved through consultation with the country coordinators under the supervision of the WHO coordinating unit.

### Data management

Trained research nurses at the participating health facilities will use paper-based data collection tools to collect data prospectively. The study coordinator in each health facility will perform a visual inspection of each form before data entry. All entries will be de-identified at the stage of data collection and participants will be identifiable only by unique identification codes that are only accessible and known to the hospital coordinator. The hospital coordinator will keep a copy of the data collection forms of each patient in a locked cabinet at the health facility until the database is considered clean and ready for final analysis. The original data collection forms of each patient will be sent to the country-level data entry center in weekly batches. All forms received at the country data entry centre will also be kept in locked cabinets accessible only to the data managers and country principal investigator until the database is ready for final analysis. Online data entry will be performed in one data-entry center from each country. These procedures have been used in previous multicentre trials and proven to be efficient and compliant with the HRP/WHO Standard Operating Procedures. Similarly, HRP has good experience with management of online data entry systems from several international multicentre studies conducted in the past 5 years [24-26]. The online data entry system also minimizes the delays in data queries and completion of incomplete forms.

RedCap, an open-source data entry system, will be used in the study. This system is being used by several institutions that conduct multicentre trials around the world. A customized data entry and monitoring system will be developed in the RedCap platform for this study. This data entry system will be password-protected and accessible only to the database managers and study team. The system will be developed and coordinated by the study Data Management Unit at the University of São Paulo, Brazil.

### Data analysis plan

A detailed plan for statistical and computational analysis will be developed by the Data Management and Analysis team in collaboration with the study coordination unit at WHO before data collection starts. This plan of analysis will be externally reviewed by an expert panel of the Bill & Melinda Gates Foundation. Modelling plan will be developed and implemented at the University of São Paulo, by a team of experts that includes biostatisticians, computational statisticians, information technology specialists and obstetricians.

A summary analysis plan is presented below by primary objective.

### Primary Objective # 1 (To identify the essential elements (including thresholds and interactions) of intrapartum monitoring that trigger the decision to use interventions aimed at preventing poor labour outcomes)

For many women, intrapartum care is composed of expectant monitoring and a supportive, hands-off approach. Other women may require interventions to avoid complications or expedite labour and delivery. During this process, health professionals are frequently acquiring information, processing it, and making the decision to keep monitoring as it is, intensifying the monitoring or intervening. We intend to mimic this process using artificial intelligence techniques and split the analysis in four phases.

#### Phase 1 – Descriptive analysis

Frequencies and proportions will be used to describe the characteristics of the study population, intrapartum care, hospital characteristics and labour outcomes.

#### Phase 2 – Determining the baseline risk

Crude and adjusted odds ratios will be used to determine the relationship between candidate predictors at hospital admission, intrapartum interventions and labour outcomes. Candidate predictors include the characteristics of women, their current and past obstetric and complications profile, the conditions of the women and the hospital capacity. Statistical and computational modeling will be used to determine the baseline risk of poor labour-related outcomes and the baseline probability of receiving selected intrapartum interventions (i.e. amniotomy, augmentation of labour, caesarean section, operative vaginal delivery). The analyses will account for clustering effect at two levels: country and hospital level.

#### Phase 3 – Determining the risk throughout labour and delivery

Each woman will have multiple data points portraying her progress during labour and delivery. At each of these data-points, the relationship between candidate predictors, intrapartum interventions and labour outcomes will be determined/updated. Statistical and computational modeling will be used to determine the baseline risk of poor labour-related outcomes and the baseline probability of receiving selected intrapartum interventions (i.e. amniotomy, augmentation of labour, caesarean section, operative vaginal delivery). These analyses will account for clustering effect at three levels: country, hospital and woman level.

In order to create trends for each variable of interest, a minimum of three measurements will be collected up to a maximum of one measurement per hour. In case of more than one measurement per hour, the assessment with the largest deviation from normality will be used. This approach was previously used to develop clinical prognostic models (e.g. Simplified Acute Physiology (SAP) and Acute Physiology and Chronic Health Evaluation (APACHE) scoring systems [27,28].

#### Phase 4 – Listing the essential elements of intrapartum monitoring (i.e. predictors of intrapartum interventions and labour outcomes)

Based on the findings of phases 1–3, in phase 4, the predictors of intrapartum interventions and labour outcomes (i.e. candidate predictors retained in the models) will be determined. These predictors will constitute the essential elements of intrapartum monitoring and action and will be included in the SELMA tool.

### Statistical and computational analysis

Intrapartum care involves critical decision points related to the use or non-use of various intrapartum interventions (e.g. augmentation, rupture of membranes, caesarean section etc.). In order to model this process, it is first necessary to identify women that are at a high risk of presenting poor intrapartum related outcomes. We will use prediction models to identify women at risk of the composite adverse outcome (and need an intervention) and use prediction models to suggest the best course of action to avert this outcome. To develop this set of prediction models we will use the best available techniques in prognostic research; including identification of important predictors, assigning relative weights to each predictor, estimation of the predictive performance of the model through calibration and discrimination, and determination of its potential for application using internal validation techniques. Only candidate predictor variables available for 80% or more of the recruited women will be included in the analyses.

In terms of modelling, we will explore four analyses techniques:

- Multilevel logistic regression. This is a standard statistical technique used in traditional prognostic research. We will use backward elimination of candidate predictors with a nominal significance level of 5%.
- Multilayer perceptron. This is an artificial neural network technique. We will use back propagation as supervised learning technique.
- Structural equation modelling. This is a statistical technique for testing and estimating causal relations using a combination of statistical data and qualitative causal assumptions.
- Support vector machines. This is a supervised learning model that analyzes data to identify patterns for classification and regression analysis.

The performance of the models derived using these techniques will be assessed for calibration and discriminatory power. Specific tests will be carried out to assess performance including calibration plots, the Hosmer-Lemeshow test, Receiver Operator Characteristics (ROC) curves/C-statistics and R square tests. For each critical node, the best performing models will be selected.

### Primary Objective # 2 (To develop a simplified, monitoring-to-action algorithm for labour management)

Clinical guidelines and algorithms depicting “global best practices” for intrapartum care exist [29,30]. The BOLD project also includes formative research (qualitative research) aimed at adapting the global best practices to the reality of intrapartum care in Nigeria and Uganda. The process of adaptation will consider the expectations, preferences and needs of women, families and communities as well as the facility-based health care providers and the capacity of local health systems. This piece of work has been submitted as a separate protocol to the WHO HRP Review Panel on Research Projects [23].

**Figure 3:**
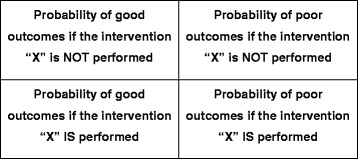
Probability panel for intrapartum decision-making (the “intervention” “X” could be: continued routine monitoring, amniotomy, augmentation of labour, caesarean section, or operative vaginal delivery).

Global best practices together with their local adaptations will form the backbone of a stepwise clinical algorithm used by SELMA. The decision points of this clinical algorithm will be fed by a network of interconnected models developed as part of the primary objective #1. Software will be developed to integrate the stepwise clinical algorithm with the interconnected mathematical models and allow input and output of information. At each decision point, and for each intervention evaluated, the probability panel showed in the Figure 3 will be calculated. Based on this probability panel, a course of action that maximizes the risk of good outcomes and minimizes the risk of poor outcomes will be suggested.

The target users for SELMA are skilled birth attendants, particularly midwives and non-specialized clinicians (i.e. clinicians without specialist training in obstetrics but who also provide care for women in labour).

### Secondary Objective # 1 *(To compare the diagnostic performance of SELMA and partograph algorithms as tools to identify women likely to develop poor labour-related outcomes)*

In the partograph, when the alert line is crossed, the woman is classified at the category of high risk of developing a poor outcome of labour. If this woman crosses the action line, she is moved to a category of very high risk of poor outcomes. Throughout labour and childbirth SELMA models will be classifying women in risk categories. These classifiers function as diagnostic tests and can be assessed as such having the final outcome as gold standard. Sensitivity, specificity, positive and negative likelihood ratios and diagnostic odds ratios will be used to compare the diagnostic performance of SELMA and the partograph.

### Ethical considerations

This is an observational study that will not expose the study participants to any additional risk. All potential participants will be approached by trained research nurses for participation at hospital admission during the early stages of labour. Women in advanced labour or who are distressed for any reason at hospital admission will not be eligible to participate as this may compromise their ability to freely and clearly decide whether they are willing to participate or not in this study. The research assistant will determine if the women are able to provide consent and will be trained to ensure voluntariness of consent. Women approached for participation will be reassured that their decision to participate will not affect the treatment they receive in the hospital. The research nurses will be trained to determine when a woman is able to provide confidential information (e.g. abortion history) that may not be available in her antenatal records. Such information will be obtained privately when there is no risk to compromising labour care (e.g. on the postnatal ward). All potential participants will receive information about the study in their language of choice, conforming to ethical requirements for research involving human subjects. The language will be easy to understand and free of technical jargons. Participants will be given sufficient time to reflect on the information and ask questions. Those who consent to participate in the study will be requested to sign the informed consent form, and it will be made clear that they are free to withdraw from the study at any stage without risk of any negative consequences. For illiterate women, an impartial witness will be present during the entire informed consent reading and discussion. Both the witness and the individual discussing the consent will sign and date the consent form. The contact details of the local investigators, including telephone numbers, will be made available to the participants should they require further information and assistance.

Participants will not experience any direct and/or immediate benefits for participating in the study. However, the study will be gathering information to inform the development of tools that have the potential to improve the quality of labour management in the future. Study participants and other women using or intending to use facilities for childbirth could indirectly benefit from the increased scientific knowledge on this topic, which will ultimately promote women-centred care of high quality in the facilities in the future. We do not anticipate any risk to individual participating woman as the participant information will remain confidential at all times and the researcher will not know the identities of the participants through the information gathered. Participants will not experience any health problems that are a direct result of participating in the study. However, should any condition be identified, the women will receive appropriate care within the health services. There will be no reimbursement or compensation provided to study participants for taking part in the study. No form of deception will be used in this study.

The WHO HRP Review Panel on Research Projects (RP2) comprising of external reviewers and WHO scientific staff reviewed and approved the scientific and technical content of the study (protocol ID, A65879). Ethics approval was obtained from the WHO Research Ethics Review Committee (ERC) and ethics review authorities responsible for all participating hospitals (Federal Capital Territory Health Research Ethics Committee and Ondo State Ministry of Health Research Ethics Review Committee in Nigeria, and Makerere School of Health Sciences Research and Ethics Committee in Uganda.

### Study timeline

This is a two-year project. It is anticipated that the preparations for this study will take approximately 9 months, recruitment into the study in the facilities can be completed in approximately 6 months and analysis can be completed in another 6 months, leaving three months for interpretation, findings reporting and dissemination and project closure.

## Discussion

### Expected outcomes

For over four decades, the partograph has been proposed as an integral part of labour management with the aim of helping to discriminate between women with normal and abnormal labour progression in all settings. However, in spite of its popularity, it is underused and the desired improvements in labour outcomes in low-resource settings remain to be seen due to several factors. This research was specifically designed to develop a tool that could overcome the challenges of making sense of labour information and customizing interventions to prevent adverse labour outcomes. It challenges and offers opportunity to revisit the theoretical basis of the partograph using up to date methodologies that have been successfully applied in other medical fields.

Based on the proposed research, we expect to develop a viable alternative to the partograph. As part of this endeavor, we expect to identify the essential elements of intrapartum monitoring, compare diagnostic performance of SELMA and partograph algorithms as tools to identify women likely to develop poor labour-related outcomes and explore the development of modern curves of normal labour progress for sub-Saharan African women.

### Main problems anticipated and proposed solutions

#### Detailed intrapartum care data is missing

If data on candidate predictors and outcomes are unavailable or insufficient, valid prognostic modeling will be impossible. In order to avoid this, the amount of data that will be collected repeatedly during labour will be limited to those traditionally recorded on the partograph. Dedicated research nurses will collect data prospectively and concurrently as it is documented by the health care providers in the case records or on the partograph; if needed, the research nurse will ask health providers for additional information.

#### Quality of intrapartum care is poor

If quality of intrapartum care is poor, the ability to generate meaningful models will be compromised. In order to avoid this, careful selection of hospitals will be performed. But precautions will be taken to select facilities that ensure external validity and allow generalizability of the results.

#### Data is insufficient to model the relationship of candidate predictors, interventions and outcomes

If data is insufficient to generate robust models, we will seek and use other data sources, such as data from the WHO Multicounty Study on Maternal and Newborn Health and from other studies that are testing the C-Models can be used to develop draft models.

### Applicability of the results

In order to maximize the applicability of its findings, this study will be conducted in two African countries, where the burden of poor outcomes related to labour and childbirth is high. Health facilities with district-level status will be selected also to maximize the applicability of the results. This project will have a second phase, where the new tools developed by the BOLD project will be adapted and tested in a large number of countries, which also favours the future applicability of results.

### Links with other projects

The development of the SELMA algorithm and tool is part of the BOLD project, a larger initiative with the overall goal of reducing adverse maternal and infant outcomes resulting from labour complications through research, design and implementation of innovative tools. The BOLD project also includes the development of a Passport for Safer Birth (PSB, another tool being developed as part of the BOLD project). The BOLD project has a qualitative, formative research component that will feed into the final development of SELMA at the implementation phase. In the future, the findings from this project may contribute to WHO guidelines on intrapartum care.

### Plans for dissemination and use of project results

The results arising from the study will be published in a reputable peer-reviewed journal. All publications will follow relevant external guidance such as the ‘*Uniform Requirements for Submission of Manuscript to Biomedical Journals*’ issued by the International Committee of Medical Journal Editors (ICMJE). Dissemination of results to participating institutions and communities will take place through meetings of stakeholders within the facilities and the communities. The results of the study will first be reported to collaborating investigators. Collaborating investigators will then disseminate local and collective results to their department and relevant authorities within the countries. There is a public website (http://www.who.int/pmnch/topics/maternal/20100914_gswch_en.pdf?ua=1) through which activities and progress of the project will be documented and shared. Additionally, a bi-monthly newsletter will be published and disseminated to all stakeholders throughout the life span of the BOLD project.

## Additional file

Additional file 1:
**Better Outcomes in Labour Difficulty Project.**
