# Supplementary material for: The development of a Simplified, Effective, Labour Monitoring-to-Action (SELMA) tool for Better Outcomes in Labour Difficulty (BOLD): study protocol
Source: Reprod Health. 2015 May 26;12:49. doi: 10.1186/s12978-015-0029-4 (PMC4448543; doi:10.1186/s12978-015-0029-4)
Supplement: Additional file 1: — Better Outcomes in Labour Difficulty Project. [file 12978_2015_29_MOESM1_ESM.pdf]

World Health Organization

BOLD

Better Outcomes in Labour Difficulty Project

INDIVIDUAL DATA COLLECTION FORM

Instructions

1. This form is composed of sections.

2. Section A MUST be completed for all women admitted for delivery.

3. If the woman is not in first stage of labour or induction of labour (Q12a=0) or if any of the other conditions is present (Q12b to Q12l equals 1), the woman is NOT eligible to participate. Informed consent is mandatory.

4. All Sections MUST be completed as applicable for ALL women included in the study

5. In case of inadequate data in the woman's case record, obtain information from attending health provider.

6. In case of erroneous entry, cross out neatly, write the correct answer outside the box and append your initials.

7. In case the data is not known, missing, or not applicable, fill in with "9", "99", or "999" (except where skip instructions are provided).

8. The use of "9", "99", or "999" is not allowed in Section F and Q74.

Data obtained through this form will help the development of a Simplified, Effective, Labour Monitoring-to-Action tool (SELMA)

This form was reviewed by the hospital coordinator and is ready for data entry	*Date*	*Hospital coordinator signature*

World Health Organization	Better Outcomes in Labour Difficulty Project<br>SELMA Development	INDIVIDUAL FORM<br>Page 1/10

A Hospital admission - All women

1) Identification:

a) Country code (N=Nigeria U=Uganda)
b) Facility code
c) Individual form number
d) Hospital ID#

2) Date of hospital admission

d d m m y y

3) Time of hospital admission (24h format) h h m m

4) Age (years)

5) Marital status
0= Married / Cohabitating
1= Single / Separated / Divorced / Widowed

6) Gravidity
(Number of pregnancies including current)

7) Parity
(Number of previous births)

8) Number of previous abortions
(includes induced and spontaneous abortions)

9) Number of previous C-Sections

10) Ethnic group

Nigeria:	Uganda:
1= Ibo	9= Muganda/Musoga/Mugisu
2= Yoruba	10= Munyakore/Mukiga/ Munyoro/Mutoro
3= Hausa	
4= Fulani	11=Acholi/Langi/Alur
5= TIV	12=Iteso/Karamojong
6= Kanuri	13=Lugbara/Madi
7= Other Nig.	14=Other Ugandan
8= Non-Nigerian	15= Non-Ugandan

11) Education level. Specify:
0= No education
1= Pre-primary education
2= Incomplete primary education
3= Complete primary education
4= Incomplete secondary education
5= Complete secondary education
6= Incomplete post-secondary/tertiary education
7= Complete post-secondary/tertiary or higher education
8= Other (e.g. Quranic / Nomadic education only)

12) Indicate whether the conditions below are present or not (0=No 1=Yes)
a) First stage of labour (spontaneous / induced)
b) Fetal death
c) Advanced first stage of labour (cervical dilation ≥7cm)
d) Multiple pregnancy
e) Gestational age less than 34 weeks
f) Elective C-section
g) Pre-labour C-section
h) Indication for emergency C-Section or laparotomy on admission
i) Attempted induction of labour, but no labour achieved
j) False labour
k) Non-emancipated minors w/o a guardian
l) Woman is not capable of giving consent due to labour distress or any health problem

> If the woman is not in labour (Q12a=0) or if any of the other conditions is present (Q12b to Q12l equals 1), the woman is **NOT** eligible to participate.

13) Does the woman consent to participate in the study? (0=No 1=Yes 9=Not eligible)

Data Collector Name:	Date:

B Hospital admission - only eligible women

14) Gainful occupation (0=No 1=Yes) ☐

15) In case of any previous abortion, specify:
(In case of no previous abortion, use 99)
a) Number of induced abortions ☐☐
b) Number of spontaneous abortions ☐☐

16) Any previous uterine surgery ☐
(0=No 1=Yes) (e.g. myomectomy)
Note: Previous CS not included

17) Antecedent history of prolonged labour / obstructed labour ☐
(0=No 1=Yes)

18) Number of previous stillbirths ☐

19) Outcome of last childbirth ☐
0= no previous childbirth
1= live birth, still alive
2= live birth, deceased
3= stillbirth

20) In case of previous childbirths, what is the date of last delivery?

d	d	m	m	y	y

21) Pre-pregnancy health conditions (0=No 1=Yes)

a) Chronic Hypertension ☐
b) Diabetes Mellitus ☐
c) HIV + ☐
d) AIDS / HIV wasting syndrome ☐
e) Chronic Anaemia (e.g. sickle cell anaemia) ☐
f) Obesity ☐
g) Heart disease ☐
h) Lung disease ☐
i) Renal disease ☐
j) Other chronic disease ☐

22) Complications in current pregnanc (0=No 1=Yes)

a) Placenta praevia ☐
b) Accreta/increta/percreta placenta ☐
c) Abruptio placentae ☐
d) Other obstetric haemorrhage ☐
e) Pre-eclampsia (excludes eclampsia) ☐
f) Eclampsia ☐
g) Pyelonephritis ☐
h) Malaria ☐
i) Preterm rupture of membranes ☐
j) Anaemia (Hct ≤ 26% or Hb ≤ 9g/dL) ☐
k) Gestational diabetes ☐
l) Other pregnancy complications ☐

23) Number of antenatal care visits ☐☐

24) Women referred in labour from another health facility (0= No 1= Yes) ☐

25) Best estimate of gestational age (in complete weeks) ☐☐

26) Method of gestational age estimation ☐
0= Symphysis-fundal height (cm)
1= Birth weight assessment
2= Last menstrual period
3= Neonatal physical findings (e.g. Ballard)
4= Ultra-sound scan

27) Mode of onset of labour ☐
0= spontaneous 1= induced

28) If spontaneous onset of labour, specify duration of labour before admission in hours ☐☐

29) Fetal movements in the last 2 hours, as self reported. ☐
(0=no changes/increased 1=reduced 2=absent)

World Health Organization

Better Outcomes in Labour Difficulty Project

SELMA Development

B Hospital admission - only eligible women (first assessment)

30) Time of first assessment (24h format) h h m m ☐☐☐☐

31) Maternal anthropometric and physical exam. findings:

- a) Current weight (kg) ☐☐☐
- b) Height (cm) ☐☐☐
- c) Foot length (cm) ☐☐
- d) Maternal heart rate ☐☐☐
- e) Systolic blood pressure (mmHg) ☐☐☐
- f) Diastolic blood pressure (mmHg) ☐☐☐
- g) Axillary temperature (℃) ☐☐☐
- h) Symphysis-fundal height (cm) ☐☐
- i) Abdominal circumference (cm) ☐☐☐
- j) Number of uterine contractions (10 min) ☐
- k) Duration of uterine contractions ☐☐
- l) Fetal heart rate (bpm) ☐☐☐
- m) Fetal movements (0=No 1=Yes) ☐
- n) Cervical dilatation (cm) (0=No 1=Yes) ☐
- o) Cervix effacement ☐
  0=Thick (<30%) 2=Thin (up to 80%)
  1=Medium (up to 50%) 3=Very thin (>80%)
- p) Cervix position ☐
  (0=anterior 1=central 2=posterior)
- q) Cervix consistency ☐
  (0=soft 1=medium 2=firm)
- r) Fetal presentation ☐
  (0= Cephalic 1=Breech 2=Transverse/other)
- s) Fetal station ☐
  0= above ischial spine
  1= at ischial spine 2= below ischial spine
- t) Position of fetal head ☐
  0= Occiput Anterior (includes right and left)
  1= Occiput transverse
  2= Occiput posterior 3= Other
- u) Caput succedaneum ☐
  0=None 1=Mild 2=Moderate 3=Severe
- v) Moulding ☐
  0= None 2= Second degree
  1= First degree 3= Third degree
- w) Amniotic membranes status ☐
  0= Intact
  1= Ruptured without meconium
  2= Rupture with stale meconium
  3= Ruptured with fresh meconium
- x) Clinical pelvic assessment
  (Specify: 0=No 1=Yes 9=Not assessed)
  1. Sacral promontory reached ☐
  2. Ischial spines prominent ☐
  3. Pubic angle admits less than 2 fingers ☐

32) To what extent has the woman been bothered by:

- a) Emotional problems? ☐
  (e.g. fear, anxiety, irritability, depression, sadness)
- b) Labour pain? ☐
  (USE THE CHART)

33) Labour companionship? (0=No 1=Yes) ☐

C Intrapartum data	Page 4/10	C1	C2	C3	C4	C5	C6
	34) Time of assessment:						
	35) Full Assessment? (0=No 1=Yes)						
36) Number of uterine contractions in 10 minutes							
37) Duration of uterine contractions (seconds)							
38) Fetal heart rate (b.p.m.)							
39) Fetal movements observed/felt (0=No 1=Yes)							
40) Cervical dilatation (cm)							
41) Fetal presentation (0=cephalic 1=breech 2=transverse lie / compound / other)							
42) Fetal station (0=above ischial spine 1= at ischial spine 2=below ischial spine)							
43) Moulding (0=None 1=First degree 2=Second degree 3=Third degree)							
44) Caput succedaneum 0=None 1=Mild 2=Moderate 3=Severe							
45) Position of fetal head 0=Occiput Anterior (includes right and left) 1=Occiput transverse 2=Occiput posterior 3=Other							
46) Amniotic membranes status 0= intact 1= ruptured without meconium 2= ruptured with stale meconium 3= ruptured with fresh meconium							
47) Systolic Blood Pressure (mmHg)							
48) Diastolic Blood Pressure (mmHg)							
49) Maternal heart rate (b.p.m.)							
50) Axillary temperature (℃, with decimals)							
51) Since the last assessment, to what extent has the woman been bothered by labour pain? (USE CHART)							
52) Since the last assessment, to what extent has the woman been bothered by emotional problems such as fear, anxiety, depression, irritability, or sadness? (USE CHART)							
53) Oxytocin infusion (0=No 1=Yes)							
54) Oxytocin dilution (0= 0.25 amp/500ml 1= 0.5 amp/500ml 2= 1 amp/500ml 3=2amp/500ml 4=Other)							
55) Oxytocin rate (drops/min)							
56) Analgesia (0=None 1=IV/IM Opioid 2=Epidural 3=Spinal 4=Other 5=Combined)							
57) Labour companionship (0=No 1=Yes)							
58) Predominant maternal position between assessments 0= upright, sitting, standing, walking, kneeing, squatting, all-4 1= recumbent, semi-recumbent, lateral, supine							
59) Oral fluid intake between assessments (0=No 1=Yes)							
60) Oral food intake between assessments (0=No 1=Yes)							
61) IV Fluids (0=No 1=Yes)							

Instructions

1. Record all full assessments (completely)
2. Between full assessments, record only abnormal values (e.g. FHR less than 120 or more than 160). This apply to FHR and maternal vital signs.
3. In full assessments, if information could not be retrieved, or was not assessed, or was missing, use 999. This only applies to full assessments.
4. Data collectors need to ensure that all information is obtained. If information is not in medical records, approach the assisting health provider or observe the pregnant wom[illegible]

C Intrapartum data

	C7	C8	C9	C10	C11	C12
34) Time of assessment:						
35) Full Assessment? (0=No 1=Yes)						
36) Number of uterine contractions in 10 minutes						
37) Duration of uterine contractions (seconds)						
38) Fetal heart rate (b.p.m.)						
39) Fetal movements observed/felt (0=No 1=Yes)						
40) Cervical dilatation (cm)						
41) Fetal presentation (0=cephalic 1=breech 2=transverse lie / compound / other)						
42) Fetal station (0=above ischial spine 1= at ischial spine 2=below ischial spine)						
43) Moulding (0=None 1=First degree 2=Second degree 3=Third degree)						
44) Caput succedaneum 0=None 1=Mild 2=Moderate 3=Severe						
45) Position of fetal head 0=Occiput Anterior (includes right and left) 1=Occiput transverse 2=Occiput posterior 3=Other						
46) Amniotic membranes status 0= intact 1= ruptured without meconium 2= ruptured with stale meconium 3= ruptured with fresh meconium						
47) Systolic Blood Pressure (mmHg)						
48) Diastolic Blood Pressure (mmHg)						
49) Maternal heart rate (b.p.m.)						
50) Axillary temperature (℃, with decimals)						
51) Since the last assessment, to what extent has the woman been bothered by labour pain? (USE CHART)						
52) Since the last assessment, to what extent has the woman been bothered by emotional problems such as fear, anxiety, depression, irritability, or sadness? (USE CHART)						
53) Oxytocin infusion (0=No 1=Yes)						
54) Oxytocin dilution (0= 0.25 amp/500ml 1= 0.5 amp/500ml 2= 1 amp/500ml 3=2amp/500ml 4=Other)						
55) Oxytocin rate (drops/min)						
56) Analgesia (0=None 1=IV/IM Opioid 2=Epidural 3=Spinal 4=Other 5=Combined)						
57) Labour companionship (0=No 1=Yes)						
58) Predominant maternal position between assessments 0= upright, sitting, standing, walking, kneeing, squatting, all-4 1= recumbent, semi-recumbent, lateral, supine						
59) Oral fluid intake between assessments (0=No 1=Yes)						
60) Oral food intake between assessments (0=No 1=Yes)						
61) IV Fluids (0=No 1=Yes)						

Instructions

1. Record all full assessments
2. Between full assessments, record only abnormal values (e.g. FHR less than 120 or more than 160). This apply to FHR and maternal vital signs.
3. In full assessments, if information could not be retrieved, or was not assessed, or was missing, use 999. This only applies to full assessments.
4. Data collectors need to ensure that all information is obtained. If iformation is not in medical records, approach the assisting health provider or observe the pregnant woma

C	Intrapartum data	Page 6/10	C13	C14	C15	C16	C17	C18
		34) Time of assessment:						
		35) Full Assessment? (0=No 1=Yes)						
36)	Number of uterine contractions in 10 minutes							
37)	Duration of uterine contractions (seconds)							
38)	Fetal heart rate (b.p.m.)							
39)	Fetal movements observed/felt (0=No 1=Yes)							
40)	Cervical dilatation (cm)							
41)	Fetal presentation<br>(0=cephalic 1=breech 2=transverse lie / compound / other)							
42)	Fetal station<br>(0=above ischial spine 1= at ischial spine 2=below ischial spine)							
43)	Moulding<br>(0=None 1=First degree 2=Second degree 3=Third degree)							
44)	Caput succedaneum<br>0=None 1=Mild 2=Moderate 3=Severe							
45)	Position of fetal head<br>0=Occiput Anterior (includes right and left) 1=Occiput transverse<br>2=Occiput posterior 3=Other							
46)	Amniotic membranes status<br>0= intact 1= ruptured without meconium<br>2= ruptured with stale meconium 3= ruptured with fresh meconium							
47)	Systolic Blood Pressure (mmHg)							
48)	Diastolic Blood Pressure (mmHg)							
49)	Maternal heart rate (b.p.m.)							
50)	Axillary temperature (℃, with decimals)							
51)	Since the last assessment, to what extent has the woman been bothered by labour pain? (USE CHART)							
52)	Since the last assessment, to what extent has the woman been bothered by emotional problems such as fear, anxiety, depression, irritability, or sadness? (USE CHART)							
53)	Oxytocin infusion (0=No 1=Yes)							
54)	Oxytocin dilution<br>(0= 0.25 amp/500ml 1= 0.5 amp/500ml 2= 1 amp/500ml 3=2amp/500ml 4=Other)							
55)	Oxytocin rate (drops/min)							
56)	Analgesia<br>(0=None 1=IV/IM Opioid 2=Epidural 3=Spinal 4=Other 5=Combined)							
57)	Labour companionship (0=No 1=Yes)							
58)	Predominant maternal position between assessments<br>0= upright, sitting, standing, walking, kneeing, squatting, all-4<br>1= recumbent, semi-recumbent, lateral, supine							
59)	Oral fluid intake between assessments (0=No 1=Yes)							
60)	Oral food intake between assessments (0=No 1=Yes)							
61)	IV Fluids (0=No 1=Yes)							

Instructions

1. Record all full assessments
2. Between full assessments, record only abnormal values (e.g. FHR less than 120 or more than 160). This apply to FHR and maternal vital signs.
3. In full assessments, if information could not be retrieved, or was not assessed, or was missing, use 999. This only applies to full assessments.
4. Data collectors need to ensure that all information is obtained. If ifomation is not in medical records, approach the assisting health provider or observe the pregnant woma

World Health Organization	Better Outcomes in Labour Difficulty Project<br>SELMA Development	INDIVIDUAL FORM<br>Page 7/10

CHART

0 - Not at all	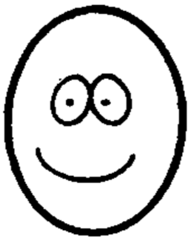
1 - Slightly	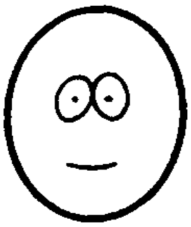
2 - Moderately	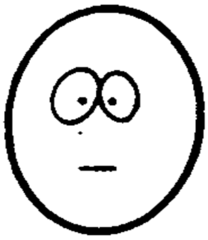
3 - Quite a bit	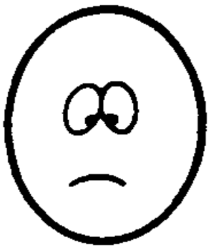
4 - Extremely	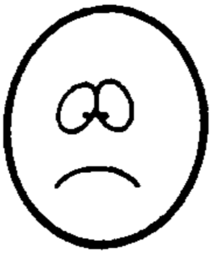

D Intrapartum interventions

62) Cervical ripening (CR) (0=No 1=Yes) ☐
(if no cervical ripening, jump to Q63)

a) Date CR starts d d m m y y ☐☐☐☐☐☐

b) Time CR starts h h m m ☐☐☐☐

c) Specify method of CR (0= No 1= Yes):

1. Oxytocin ☐
2. Misoprostol ☐
3. Other prostaglandin ☐
4. Sweep membranes ☐
5. Amniotomy ☐
6. Mechanical (e.g. Foley Catheter) ☐

63) Induction of labour (IOL) (0=No 1=Yes) ☐
(If no induction of labour, jump to Q64)

a) Date IOL starts d d m m y y ☐☐☐☐☐☐

b) Time IOL starts h h m m ☐☐☐☐

c) IOL indication (0=No 1=Yes)

1. Suspected fetal growth impairment ☐
2. Prelabour rupture of membranes ☐
3. Chorioamnionitis ☐
4. Pre-eclampsia/eclampsia ☐
5. Gestational ≥ 41 weeks ☐
6. Any other obstetric complication ☐
7. Any other medical complication ☐
8. Maternal request ☐
9. IOL without medical reason ☐

World Health Organization	**Better Outcomes in Labour Difficulty Project**<br>**SELMA Development**	**INDIVIDUAL FORM**<br>**Page 8/10**

D Intrapartum interventions (Cont'd)

d) Specify method of induction (0= No 1= Yes):
1. Oxytocin ☐
2. Misoprostol ☐
3. Other prostaglandin ☐
4. Sweep membranes ☐
5. Amniotomy ☐
6. Mechanical (e.g. Foley Catheter) ☐

64) Augmentation of labour (0=No 1=Yes) ☐
(If no augmentation of labour, jump to Q65)

a) Time of decision h h m m

b) Time infusion starts h h m m

c) Indication ☐
0= weak/irregular contractions with slow progress
1= weak/irregular contractions w/o slow progress
2= other

65) Amniotomy (0=No 1=Yes) ☐
(If no amniotomy, jump to Q66)

a) Date of amniotomy d d m m y y

b) Time of amniotomy h h m m

c) Indication ☐
0= accidental rupture
1= routine rupture
2= slow progress

d) Complication ☐
0= No
1= Cord prolapse
2= Fetal distress
3= Other

66) Caesarean section (CS) ☐
0= not performed and not decided on
1= not performed but decided on
2= performed

(If no Caesarean section, jump to Q67)

a) Time of decision h h m m

b) Time of incision h h m m

Conditions preceeding the CS:
0= Fetal distress
1= Prolonged labour
2= Cephalopelvic disproportion
3= Obstruction
4= Suspected/imminent uterine rupture
5= Failed operative vaginal delivery
6= Pre-eclampsia/eclampsia
7= Gestational ≥ 41 weeks
8= Intrapartum vaginal bleeding
9= Breech or other malpresentation
10= Previous CS
11= Failed induction
12= Maternal request
13= HIV
14= Genital Herpes/extensive condyloma
15= Previous uterine surgery
16= Other complication

c) Primary indication of C-Section ☐☐

d) Secondary indication of C-Section ☐☐

World Health Organization	Better Outcomes in Labour Difficulty Project<br>SELMA Development	INDIVIDUAL FORM<br>Page 9/10

E Childbirth and Neonatal Outcomes

67) Date of delivery d d m m y y

68) Time of delivery h h m m

69) Final mode of delivery. Specify:
0= Spontaneous vaginal delivery without episiotomy
1= Spontaneous vaginal delivery with episiotomy
2= Operative vaginal delivery (forceps or vacuum)
3= Caesarean section
4= Laparotomy

70) Fetal presentation at delivery
0=Cephalic 1=Breech 2=Transverse Lie / Other

71) Infant sex (0=Male 1=Female)

72) Birth weight (g)

73) Neonatal conditions at birth
a) Vital status (0= Alive 1= Stillbirth)
b) Apgar score at 5 min

74) Neonatal severe morbidity. Specify (0=No 1=Yes)

a) Admission to neonatal ICU/special care unit
b) Any intubation (at birth or during the 1st wk)
c) Nasal CPAP
d) Surfactant administration
e) Cardio-pulmonary resuscitation
f) Use of any vasoactive drug
g) Use of anticonvulsants
h) Use of phototherapy in the first 24 hours
i) Use of any blood products
j) Use of steroids to treat hypoglycaemia
k) Use of therapeutic intravenous antibiotics
l) Any surgery
m) Any severe malformation

75) Newborn status at hospital discharge
(0= Alive 1=Dead)

76) Newborn discharge, transfer or death
a) Date d d m m y y
b) In case of newborn death (Q75=1) what is the time of newborn death? h h m m

F Maternal Outcomes

77) Was any of the following conditions identified during hospital stay? (0=No 1=Yes)

Dystocia
a) Labour obstruction
b) Prolonged first stage of labour
c) Prolonged second stage

Haemorrhage
d) Placenta praevia
e) Accreta/increta/percreta placenta
f) Abruptio placenta
g) Ruptured uterus
h) Intrapartum haemorrhage
i) Postpartum haemorrhage

Infection
j) Puerperal endometritis
k) Puerperal sepsis
l) Wound infection
m) Systemic infection / septicaemia

Hypertension
n) Severe hypertension
o) Pre-eclampsia (excludes eclampsia)
p) Eclampsia

Other maternal complications
q) Embolic disease
r) Other potentially life-threatening condition

F Maternal Outcomes

78) Was any of the following conditions identified?

0= No

1= Yes, **within** 24h of hospital stay

2= Yes, **after** 24h of hospital stay

a) Cardiovascular dysfunction ☐

Shock

Cardiac Arrest

Severe hypoperfusion (lactate >5 mmol/L or >45mg/dL)

Severe acidosis (pH<7.1)

Use of continuous vasoactive drugs

Cardio-pulmonary resuscitation

b) Respiratory dysfunction ☐

Acute cyanosis

Gasping

Severe tachypnea (respiratory rate>40 breaths per minute)

Severe bradypnea (respiratory rate<6 breaths per minute)

Severe hypoxemia

(O2 saturation <90% for ≥60min or PAO2/FiO2<200)

Intubation and ventilation not related to anaesthesia

c) Renal dysfunction ☐

Oliguria non responsive to fluids or diuretics

Severe acute azotaemia

(creatinine >300umol/ml or >3.5mg/dL)

Dialysis for acute renal failure

d) Coagulation dysfunction ☐

Failure to form clots

Severe acute thrombocytopenia (<50,000 platelets/ml)

Massive transfusion of blood or red cells (≥ 5 units)

e) Hepatic dysfunction ☐

Jaundice in the presence of pre-eclampsia

Severe acute hyperbilirubinemia

(bilirubin>100umol/L or >6.0mg/dL)

f) Neurologic dysfunction ☐

Prolonged unconsciousness or coma (lasting >12 hours)

Stroke or Global paralysis

Uncontrollable fit / status epilepticus

g) Uterine dysfunction ☐

Hysterectomy due to uterine infection or haemorrhage

79) Please specify whether the women used any of the following interventions (0=No 1=Yes)

Haemorrhage

a) Oxytocin for treatment of PPH ☐

b) Misoprostol for treatment of PPH ☐

c) Ergotamine for treatment of PPH ☐

d) Other uterotonic for treatment of PPH ☐

e) Artery ligation or embolization ☐

f) Balloon or condom tamponade ☐

g) Repair of cervical laceration ☐

h) Repair of uterine rupture ☐

i) B-lynch suture ☐

j) Hysterectomy ☐

Infection

k) Therapeutic antibiotics (excludes prophylaxis) ☐

Hypertension

l) Magnesium sulphate as anticonvulsant ☐

m) Other anticonvulsant for eclampsia ☐

Other interventions

n) Removal of retained products ☐

o) Manual removal of placenta ☐

p) Blood transfusion ☐

q) Laparotomy ☐

r) Admission to Intensive Care Unit ☐

80) Specify whether the woman was referred to any higher complexity hospital (0=No 1=Yes) ☐

81) Maternal status at hospital discharge (0= Alive 1=Dead) ☐

82) Date of maternal discharge, transfer or death

d	d	m	m	y	y

The use of "9" is not allowed in section F and Q74

Data Collector Name: **Date:**
